# Dimensioning of painful procedures and interventions for acute pain relief in premature infants ^1^

**DOI:** 10.1590/1518-8345.1387.2917

**Published:** 2017-09-18

**Authors:** Deise Petean Bonutti, Mariana Firmino Daré, Thaíla Corrêa Castral, Adriana Moraes Leite, Joselaine Aparecida Vici-Maia, Carmen Gracinda Silvan Scochi

**Affiliations:** 2 MSc, RN, Hospital das Clínicas, Faculdade de Medicina de Ribeirão Preto, Universidade de São Paulo, Ribeirão Preto, SP, Brazil.; 3 Doctoral student, Escola de Enfermagem de Ribeirão Preto, Universidade de São Paulo, PAHO/WHO Collaborating Centre for Nursing Research Development, Ribeirão Preto, SP, Brazil. Scholarship holder at Fundação de Amparo à Pesquisa do Estado de São Paulo (FAPESP), Brazil.; 4 PhD, Adjunct Professor, Faculdade de Enfermagem, Universidade Federal de Goiás, Goiânia, GO, Brasil.; 5 PhD, Associate Professor, Escola de Enfermagem de Ribeirão Preto, Universidade de São Paulo, PAHO/WHO Collaborating Centre for Nursing Research Development, Ribeirão Preto, SP, Brazil.; 6 RN, Hospital das Clínicas, Faculdade de Medicina de Ribeirão Preto, Universidade de São Paulo, Ribeirão Preto, SP, Brazil.; 7 PhD, Full Professor, Escola de Enfermagem de Ribeirão Preto, Universidade de São Paulo, PAHO/WHO Collaborating Centre for Nursing Research Development, Ribeirão Preto, SP, Brazil.

**Keywords:** Acute Pain, Critical Pathways, Infant, Premature, Infant, Newborn, Pain Management, Neonatal Nursing

## Abstract

**Objective:**

to dimension the exposure of premature infants to painful procedures, relating the distribution of the exposure to contextual factors, as well as to describe the pharmacological and non-pharmacological interventions health professionals use during the first two weeks of the infant’s hospitalization at two neonatal services.

**Method:**

descriptive-exploratory study in which the professionals registered the painful procedures and pain relief interventions on a specific form in the patient file.

**Results:**

the daily average of the 89 premature infants was 5.37 painful procedures, corresponding to 6.56 during the first week of hospitalization and 4.18 during the second week (p&lt;0.0001). The most frequent procedures were nasal/oral (35.85%) and tracheal aspirations (17.17%). The children under invasive ventilation were the most exposed to painful procedures (71.2%). Only 44.9% of the painful procedures received some intervention for the purpose of pain relief, the most frequent being sucrose (78.21%) and continuing sedation (19.82%).

**Conclusion:**

acute pain was undertreated at these neonatal services, recommending greater sensitization of the team for the effective use of the existing protocol and implementation of other knowledge transfer strategies to improve neonatal pain management.

## Introduction

Preterm birth has represented a public health problem, which requires specific interventions to improve this population’s quality of life^1^. Preterm infants were born before the 37^th^ week of pregnancy^1^, classified as extremely preterm (&lt;28 weeks of pregnancy), very preterm (between 28 and &lt;32 weeks of pregnancy) and moderate to late preterm (between 32 and &lt;37 weeks of pregnancy)^1^.

The preterm infant’s trajectory starts with the hospitalization, often over long periods, at neonatal services, where they are exposed to many stimuli related to lighting, noise and manipulation^2^.

Among the different types of manipulation the preterm infants are exposed to, the painful procedures stand out, which are necessary for the sake of diagnostic and therapeutic implementation, causing immediate changes in physiological (such as cardiac frequency, respiratory frequency, blood pressure, oxygen saturation and hormone dosage) and behavioral parameters (such as crying, motor activity, facial mimics, state of irritability and agitation)^3^ and, in the long term, triggering phenomena such as allodynia and hyperalgesia^4^.

Therefore, the exposure of infants to painful procedures has been investigated in different countries (such as the Netherlands, Australia, Canada and France), using different methodological designs to dimension the exposure of full-term and preterm infants and children to painful procedures during 24 hours^5^, 14 days of hospitalization^6^ ^-^ ^7^ ^)^ or 28 days or longer^8^, at a Neonatal Intensive Care Unit (UTIN). Recently, only one study has been identified in Brazil that dimensioned the pain of infants during seven days at an UTIN^9^. The exposure to painful procedures differs among the studies, ranging between treatment and 14 painful procedures per day^5^ ^-^ ^9^.

Specifically preterm infants are more vulnerable to the effects of repeated and prolonged exposure to painful and stressing procedures, which can contribute to neurobehavioral development problems^10^. It is important to study this specific population concerning the exposure to painful procedures and acute pain management by the health professionals at neonatal services.

In addition, contextual factors related to the individual (maturity, health condition and temperament), the environment (diagnostic and therapeutic interventions) and the care team (positioning and manipulation) can influence the pain response^11^.

Strong scientific evidence exists for the use of pharmacological^12^ and non-pharmacological interventions, such as sucrose^13^, kangaroo position^14^ and breastfeeding^15^, on neonatal pain reduction, but the long-term effects of these interventions remain unknown.

Thus, this study is justified by the need for deeper knowledge on the exposure of preterm infants to painful procedures and pain reduction interventions. The objective is to dimension the exposure of premature infants to painful procedures in terms of type, quantity and frequency, during the first two weeks of hospitalization at two neonatal services, relating the distribution of the exposure to contextual factors (length of hospitalization, weight at birth, gestational age, sex, clinical risk, first and fifth-minute Apgar score and ventilation support); and to describe the pharmacological and non-pharmacological interventions the health professionals use for acute pain relief during that period.

## Method

A descriptive-exploratory study carried out in the Neonatal Intensive Care Unit (UTIN) and Neonatal Intermediary Care Unit of a school hospital in the interior of São Paulo, with a total of 47 beds, with occupancy rate of 78.4% and admission rate of 73.9%. The inclusion of these services is justified because, despite different levels of complexity, they represent continuity of care to the preterm, following the same pain management protocols.

The study is registered on the Brazil platform, under CAAE Protocol 04221612.9.0000.5393 and was approved by the Ethics Committee in Research with Human Subjects, under Opinion 144/2012. The parents of the preterm infants included in the study consented to participate through the Informed Consent Form (TCLE), signed prior to data collection.

Premature infants admitted up to the third day of life in the UTIN of the hospital were followed up for 14 days, even if transferred to the Neonatal Intermediary Care Unit of the same institution, since the services, even with different levels of complexity, guarantee the continuity of care, and those who were discharged from hospital, transferred to another institution or who died before completing the 14 days of hospitalization were excluded.

The study was developed in two stages. In the first one, the health professionals, who worked in direct care for the preterm and performed painful procedures, registered the painful procedures performed in a questionnaire adapted and validated for Portuguese^6^ daily, during the first 14 days of hospitalization of the infant. The questionnaire was later attached to the medical record of the preterm infant. It consisted of 24 lines that indicated the time of day, while the columns contained information about the type of procedure, type of intervention for pain relief, the number of attempts, the category of the professional who performed the procedure and the presence of the parents in the neonatal unit during the procedure. On the back of the questionnaire was a caption for each item to be completed, which was adapted according to the context of the services, the medications used for analgesia and sedation and the professional categories that worked in direct care for the infant. The researcher and a research assistant trained the health professionals of the neonatal services regarding the research objectives and correct completion of the questionnaire. Each day, the researcher or a research assistant replenished the questionnaire in the medical record.

In the second phase, the researcher and a research assistant carried out the search of the preterm infants’ records to transcribe the procedures recorded in the questionnaire and complement the data with other procedures not registered by the professionals, but identified from the printed sheets attached to the medical record, such as an admission record in the unit, prescription and medical evolution sheets, history and nursing diagnosis, physiological data control sheets, control of laboratory tests, prescriptions, evolutions and nursing notes. In addition to painful procedures and interventions for pain relief, data on birth (date of birth, sex, weight, Apgar and gestational age) and clinical condition (major medical diagnoses, clinical risk score, use of continuous or intermittent analgesic medications) were collected from the files.

Data collection occurred between October 1, 2013 and March 31, 2014. Of the 238 newborns admitted to the unit, 176 were preterm, but only 99 preterm infants met the study criteria. There was a loss of 10 participants, due to the inaccessibility of their records in the second phase of data collection, leaving 89 participants.

Double data entry was executed and the Statistical Package for the Social Sciences (SPSS) software was used. In the quantitative analysis, descriptive statistics were used. The distribution of painful procedures was tested by the Kolmogorov-Smirnov test (α = 0.05) and, in order to compare the mean numbers of painful procedures, according to the variables “birth weight” and “gestational age”, one-way ANOVA (α = 0.05) was applied; for “sex” and “invasive ventilation”, the unpaired Student t test (α = 0.05) and “hospitalization period”, Student’s t test for two paired samples (α = 0.05).

## Results

Of the 89 preterm infants in the study, 46 (51.7%) were males. The mean weight at birth was 1,384.1 ± 615.7 grams; 27 (30.3%) neonates weighed less than 1,000 grams, 28 (31.5%) between 1,000 and 1,499 grams and 34 (38.2%) had a weight greater than or equal to 1,500 grams. The mean gestational age at birth was 30.6 ± 3.1 weeks, presenting the following distribution according to gestational age: 20 (22.5%) with gestational age less than 28 weeks, 43 (48, 3%) between 28 and 32 weeks and 26 (29.2%) more than 32 weeks of pregnancy. The mean Apgar in the 1st and 5th minutes of life was 5.5 ± 2.7 and 8.2 ± 1.8, respectively. The Score for Neonatal Acute Physiology-Perinatal Extension II (SNAPPE II) ranged from 0 to 83, with a mean of 25.8 ± 20.2.

The most frequent clinical diagnoses recorded in patient histories were: early respiratory distress (41.6%), hyaline membrane disease (29.2%), infectious risk (21.3%), maternal drug abuse (11.2% (3.4%), congenital syphilis (3.4%), neonatal sepsis (3.4%), seizures (3.4%), meningomyelocele (2, 2%) and fetal anemia (2.2%); and other diagnoses recorded in only one of the participants (1.1%): pneumothorax, intestinal dilation, Hirschsprung’s disease, enterocolitis, ambiguous genitalia, congenital pneumonia, pericardial effusion, tetralogy of Fallot and ascetic abdomen. It should be emphasized that each preterm infant can present more than one diagnosis.

During the study period, 86 (96.6%) preterm infants required oxygen therapy, with or without ventilation support, 30 (33.7%) preterm infants remained ventilated during the whole period (mechanical ventilation and / or Continuous Positive Airway Pressure - CPAP) and only 3 (3.4%) remained in ambient air during the 14 days of hospitalization.

The length of stay in UTIN ranged from 1 to 247 days, with an average hospitalization time of 28.9 ± 37.7 days. In the Neonatal Intermediary Care Unit, the hospitalization time varied between 0 and 114 days, obtaining an average of 27.2 ± 22.7 days of hospitalization. The mean length of hospital stay was 56.1 ± 50.4 days.

As for painful procedures, the 89 preterm infants were exposed to 6,678 painful procedures, with 12,300 attempts, during the first two weeks of life in the neonatal units, averaging 1.8 ± 1.0 attempts per procedure. Each preterm infant was exposed to an average of 75.1 ± 42.6 painful procedures during the 14 days, resulting in a daily average of 5.4 ± 4.9 procedures per preterm. The painful procedures performed and their frequency are presented in Table 1.

**Table 1:** Painful procedures undertaken in preterm infants during 14 days of hospitalization at neonatal services. Ribeirão Preto, SP, Brazil, 2012/2013

**Procedures**	f*	**%**
**Oral or nasal aspiration**	**2,397**	**35.85**
**Tracheal aspiration**	**1,445**	**21.61**
**Adhesive removal**	**1,148**	**17.17**
**Arterial puncture**	**573**	**8.57**
**Venipuncture**	**501**	**7.49**
**Passing of gastric/enteral tube**	**206**	**3.08**
**Heel lance**	**143**	**2.14**
**Passing of PICC** ^**†**^	**58**	**0.87**
**Tracheal intubation**	**53**	**0.79**
**Tracheal extubation**	**50**	**0.75**
**Insertion of umbilical catheter**	**32**	**0.48**
**Others** ^**‡**^	**24**	**0.36**
**Passing of urinary catheter**	**12**	**0.18**
**Phlebotomy**	**7**	**0.10**
**Lumbar puncture**	**7**	**0.10**
**Rectal stimulus** ^**§**^	**6**	**0.09**
**Intramuscular injection**	**6**	**0.09**
**Chest drain insertion**	**5**	**0.07**
**Chest drain removal**	**5**	**0.07**
**Intestinal wash through stoma** ^**§**^	**5**	**0.07**
**Subcutaneous injection**	**2**	**0.03**
**Dilated eye exam**	**1**	**0.02**
**Abdominal puncture** ^**†**^	**1**	**0.02**
**Total**	**6,687**	**100**

*f=absolute frequency; %=percentage; ^†^PICC=peripherally inserted central catheter; ^‡^procedures registered by professionals in the questionnaire by completing the space “others”, but without specifying the procedure; ^§^procedures registered by professionals in the questionnaire by completing the space “others”

Table 2 shows that, on the second day, a larger number of procedures (703) took place, with a mean of 7.90 ± 3.51, and a gradual decrease thereafter. It should be noted that the first day does not imply the first 24 hours of life, since the questionnaire was placed in the chart at the time of admission and changed at 0h. Therefore, if the premature infant was admitted to the unit at night, the second day started few hours later.

**Table 2:** Painful procedures applied per day to preterm infants during 14 days of hospitalization at neonatal services. Ribeirão Preto, SP, Brazil, 2012/2013

**Days of hospitalization**	**Number of procedures**	X^*^ **procedures per infant**	**σ** ^**†**^
**1** ^**st**^	**543**	**6.10**	**2.93**
**2** ^**nd**^	**703**	**7.90**	**3.51**
**3** ^**rd**^	**621**	**6.98**	**3.38**
**4** ^**th**^	**597**	**6.71**	**3.65**
**5** ^**th**^	**566**	**6.36**	**3.85**
**6** ^**th**^	**544**	**6.12**	**4.04**
**7** ^**th**^	**509**	**5.72**	**4.16**
**8** ^**th**^	**451**	**5.07**	**3.95**
**9** ^**th**^	**426**	**4.79**	**4.49**
**10** ^**th**^	**375**	**4.21**	**4.15**
**11** ^**th**^	**362**	**4.08**	**3.70**
**12** ^**th**^	**358**	**4.03**	**4.49**
**13** ^**th**^	**333**	**3.74**	**3.78**
**14** ^**th**^	**299**	**3.76**	**3.96**
**Total**	**6,687**	**5.37**	**4.09**

*X=mean; †σ=standard deviation

When comparing the exposure of preterm infants to painful procedures, from the first to the second week, in total, 4,082 procedures happened during the first week and 2,605 painful procedures during the second, with a daily average per preterm infant of 6.56 ± 0.72 and 4.18 ± 0.51, respectively. There was a statistically significant decrease in exposure to painful procedures in the second week (paired Student t-test p &lt;0.0001), that is, in the second week of hospitalization, the preterm infants were less exposed to pain.

The distribution of the number of painful procedures according to the birth weight range of the 89 preterm participants in the study was 2,831 (42.3%) in preterm infants weighing up to 1,000 g, 1,931 (28.9%) in preterm infants weighing between 1,001 and 1,500 g and 1,925 (28.8%) in preterm infants weighing more than 1,500 g. When comparing the averages of painful procedures per infant during the 14 days of data collection, there was a statistically significant difference (ANOVA one-way p &lt;0.0001), with a higher exposure of preterm infants with birth weight up to 1,000 g, that is, the lower the birth weight, the greater the exposure of premature infants to painful procedures.

The distribution of painful procedures according to gestational age corresponded to 2,059 (30.8%) procedures in children younger than 28 weeks of gestational age, 3,145 (47.0%) painful procedures in preterm infants of 28 to 32 weeks of gestational age and 1,486 (22.2%) in infants with more than 32 weeks of gestational age. When comparing the means of painful procedures per preterm infant during the 14 days of data collection, a statistically significant difference (ANOVA one-way p &lt;0.0001) was observed, with a higher exposure of those younger than 28 weeks, that is, the exposure to pain increases with the reduction of maturity.

The distribution of painful procedures according to the sex of the infant was 2,809 (42.0%) of the painful procedures in the female sex and 3,378 (58.0%) in the male sex. When comparing the means, there was a statistically significant difference (unpaired Student’s t-test p &lt;0.029): the male sex presented a higher mean number of painful procedures (84.30 ± 42.03) than the female sex (65.33 ± 38.12).

When analyzing the distribution of painful procedures, according to the categories of the clinical risk scale SNAPPE II, it is verified that the two premature infants with a score ≥80 were submitted to 202 (3.0%) painful procedures, that is, 101 procedures per infant during the 14 days of hospitalization while, in those 21 premature infants with a score from 0 to 9, the number of painful procedures was 1,005 (15.0%), representing 48 procedures per infant. Thus, a greater number of painful procedures per infant are observed in the category that represents greater clinical risk, that is, preterm infants a more severe clinical status are more exposed to painful procedures.

Regarding the distribution of painful procedures, according to the use of ventilation support, there were more procedures among preterm infants who used mechanical ventilation during the first 14 days of hospitalization in the neonatal services and were exposed to 1,957 (29.3%) painful procedures, followed by those submitted to ventilation and CPAP with 1,366 (20.4%) procedures, as shown in Table 3.

**Table 3:** Distribution of preterm infants’ painful procedures according to use or not of ventilation support during 14 days of hospitalization at neonatal services. Ribeirão Preto, SP, Brazil, 2012/2013

**Ventilation support**	**No. of procedures**	**%**
**No ventilation support (ambient air)**	**92**	**1.4**
**Mechanical ventilation**	**1,957**	**29.3**
Mechanical ventilation and CPAP*	**1,366**	**20.4**
Mechanical ventilation, ambient air and CPAP*	**1,032**	**15.2**
Ambient air and CPAP*	**658**	**9.8**
Mechanical ventilation, ambient air, oxygen therapy and CPAP*	**567**	**8.5**
Ambient air, oxygen therapy and CPAP*	**436**	**6.5**
**Mechanical ventilation and ambient air**	**278**	**4.2**
Mechanical ventilation, oxygen therapy and CPAP*	**163**	**2.4**
CPAP*	**62**	**0.9**
**Ambient air and oxygen therapy**	**48**	**0.7**
**Mechanical ventilation, ambient air and oxygen therapy**	**28**	**0.4**
**Total**	**6,687**	**100**

%=percentage; *CPAP=Continuous Positive Airway Pressure

Regarding whether or not invasive ventilation was used, preterm infants who used IMV (Intermittent Mandatory Ventilation) or SIMV (Synchronized Intermittent Mandatory Ventilation) were exposed to 4,762 (71.2%) painful procedures, followed by those who did not use invasive ventilation, with exposure to 1,296 (19.4%) painful procedures, and use of high-frequency ventilation, with exposure to 629 (9.4%) painful procedures during 14 days of hospitalization at the neonatal services. When comparing the use of invasive ventilation (IMV, SIMV and / or high frequency), the 33 premature infants who had not used this ventilation had an average exposure of 39.27 ± 18.67 painful procedures, while the 56 preterm infants who received this ventilation received an average 96.27 ± 35.65 (1.71%) of painful procedures, a statistically significant difference (unpaired Student t test p = 0.0001), that is, premature infants who used invasive ventilation were more exposed to painful procedures.

When comparing preterm infants who spent the 14 days on invasive ventilation (n = 15) with those who stayed the same period in ambient air (n = 3), the total was 1,957 (mean = 130.5 ± 62, 8) and 92 (mean = 30.7 ± 8.3), respectively, that is, premature infants who underwent invasive ventilation received a proportionally higher number of painful procedures compared to those in ambient air.

Of the 6,687 painful procedures, only 3,002 (44.9%) were followed up with some (pharmacological or non-pharmacological) intervention for pain relief. When analyzing the interventions used during the first 14 days of hospitalization of the preterm infants, there was a higher frequency of oral sucrose, used in 2,348 (78.21%) painful procedures, while other measures were used only once (0.03%) each - dose of midazolam, breastfeeding, local sedation, human milk and containment, as presented in Table 4.

**Table 4:** Distribution of painful procedures accompanied by some type of acute pain relief intervention in preterm infants during 14 days of hospitalization at the neonatal services. Ribeirão Preto, SP, Brazil, 2012/2013

**Interventions**	f*	**%**
**Oral sucrose**	**2,348**	**78.21**
**Continuing sedation**	**595**	**19.82**
**Fentanyl (dose)**	**38**	**1.27**
**Oral sucrose + NNS** ^**†**^	**9**	**0.30**
**SNN** ^**†**^	**5**	**0.17**
**Skin-to-skin**	**2**	**0.07**
**Midazolam (dose)**	**1**	**0.03**
**Breastfeeding**	**1**	**0.03**
**Local sedation**	**1**	**0.03**
**Human milk**	**1**	**0.03**
**Containment**	**1**	**0.03**
**Total**	**3,002**	**100**

n=3,002; *f=absolute frequency; %=percentage; ^†^NNS=non-nutritive sucking

## Discussion

In total, there were 6,687 painful procedures, with a daily average of 5.37 procedures per premature, a value lower than that found in previous studies conducted in other countries, with a similar methodological design.

In France^6^, 430 newborns received 42,413 painful procedures in the first 14 days in UTIN, totaling the daily average of 12 procedures per newborn. In one UTIN in the Netherlands^7^, 151 newborns received 19,674 painful procedures in 14 days, with an average of 14 procedures per day per newborn. Eight years later^16^, in the same Dutch UTIN, 175 newborns received 21,076 painful procedures in 14 days, with an average of 11.4 daily procedures per newborn.

It should be noted that, despite similar methods - registers in medical records or in forms by professionals delivering care to infants - the studies included the full-term population requiring UTIN, which usually presents diseases such as congenital heart disease, anoxia, malformation and other pathologies, which can greatly increase the number of painful procedures. In addition, these studies come from developed countries, which have greater technological contribution to survival, implying greater exposure to painful procedures.

In Brazil^9^, 32 newborns in intensive care and medium-complexity neonatal intermediary care were submitted to 1,316 painful procedures, recorded on a specific form during seven days of hospitalization, with a daily average of 5.9 procedures per newborn. Similar data were found in the retrospective review of records of 32 pediatric and neonatal units in Canada, identifying that 78.2% of the 3,822 children studied underwent at least one painful procedure in 24 hours, with a mean of 6.3 painful procedures per child^5^.

In the studies with data from records, whether using a specific questionnaire^6^ ^-^ ^7^ ^,^ ^9^ ^,^ ^16^ or the institution’s printed form^5^, like in the present study, the limitation of possible underreporting of painful procedures should be considered.

Regarding birth weight, based on the classification of extreme low weight (less than 1,000 g), very low weight (less than 1,500 g), and according to the distribution of birth weights of preterm infants included in the study, the sample was classified in categories of weight up to 1,000g, from 1,001g to 1500g and greater than 1,500g. The results showed greater exposure to painful procedures in premature infants who presented lower birth weight, that is, in extremely low birth weight preterm infants.

The same occurs with gestational age: it is observed that, the more immature the newborn’s classification, the greater the exposure to pain. Thus, the extreme premature infants, in addition to the biological risk inherent to maturity, are subject to the harmful consequences of repeated exposure to painful procedures^10^.

The findings of painful procedures, in relation to weight and gestational age, corroborate a study^6^ where a statistically significant difference (p &lt;0.001) was observed in the logistic regression model, comparing the exposure to painful procedures, associated with the use of sedation in newborns with gestational age between 37 and 42 weeks compared with the categories of preterm infants. It should be noted that, in the present study, painful procedures were not evaluated regarding the relationship between weight and gestational age (if birth weight for gestational age is adequate), which is a gap to be explored in future studies.

In the neonatal units studied, it is part of the protocols that clinical risk is assessed using SNAPPE II. When analyzing the distribution of painful procedures according to SNAPPE II, in the score range ≥80, which represents a risk of 63.8% or more^17^, there was a greater exposure to painful procedures due to prematurity (101), that is, there is an indication that preterm infants in worse clinical condition receive more painful procedures. In addition, the preterm infants who spent the 14 days of data collection on invasive ventilation presented a proportionally higher number of painful procedures compared to those who spent the same period in ambient air, confirming study findings with a similar design^6^. Those preterm infants who have a more aggravated state of health and need greater procedural interventions to maintain survival are consequently more exposed to acute pain and its repeated effects.

Like in the results of this study, others also reported that the most frequent painful procedures performed in neonatal services are nasal / oral aspiration, followed by tracheal aspiration^6^ ^-^ ^7^. This is justified by the fact that the studies are carried out in intensive care units, with a large number of infants requiring mechanical ventilation. In another study, developed at a unit of lower complexity^9^, the most frequent procedure was heel lance, a procedure that ranked seventh in this study. This difference is due to the fact that, in the neonatal units in this study, venous and arterial blood collections were used for glucose measuring, according to the care planning, as a strategy to reduce the number of painful procedures in neonates at risk and as a neonatal pain management measure, as the heel lance is a more painful procedure^18^.

In the 32 investigated Canadian pediatric and neonatal units^5^, the most frequently performed procedures in terms of pain intensity were: moderate to severe venipuncture and peripheral catheter insertion; mild to moderate - heel lance, endotracheal suction, fixation exchange / removal, mobilization, endotracheal tube removal and gastric tube insertion; and mild - oral or nasal aspiration, peripheral intravenous catheter removal, urinary catheter and gastric tube removal.

Regarding the insertion of PICC and umbilical catheter, it is observed that these procedures are very infrequent (0.87 and 0.48%), although they are longer catheters and avoid arterial and venous punctures. Therefore, its use should be encouraged, and protocols based on specific evidence should be created in order to reduce manipulation and exposure to painful procedures.

As for the use of interventions for pain management, the under-treatment of pain is noted, since most of the painful procedures (55.1%) were performed without the use of interventions for pain relief. Other studies in the international scenario also picture the under-treatment of neonatal pain^5^ ^-^ ^6^, although not in majority percentages like in this study. The possible underreporting of painful procedures and the use of interventions, even with implanted clinical protocols, should be pointed out as a research limitation, including in this study.

It is worth remembering, however, that the proper management of pain is considered a fundamental human right. Thus, it becomes a right of all people to have access to proper pain management without any discrimination, to acknowledge patients’ pain and to provide information to them on the means available to evaluate and treat it, as well as access to appropriate evaluation and treatment by trained health professionals^19^. In Brazil, the right not to feel pain is reserved to the child by way of Resolution 41/95 - Rights of Hospitalized Children and Adolescents^20^, and by the Universal Declaration of Rights for Premature Infants^21^.

In the present study, among the interventions used, the most frequent was the oral sucrose solution. There are about 80 clinical trials and literature reviews and a systematic review on the use of this sweetened solution^13^. In a systematic review^22^, it was concluded that doses of 0.5 to 2 ml of sucrose (12 to 50%) administered orally two minutes before the painful procedure, combined with non-nutritive suction, reduce 1-2 points in the pain scale. In another review^23^, however, doubts are raised about the analgesic properties of sucrose, since the administration of this solution reduced the external manifestations of pain in newborns, such as facial movements, crying, heart rate and pain scores on single and multidimensional scales, when offered before acute painful procedures like the heel lance; some children showed cortical responses though, even without changes in facial expression. These reflections have raised the possibility that reduced behavioral activity may not mean effective sedation, in addition to raising the issue of hyperalgesia (increased sensitivity to subsequent painful events) as a consequence of exposure to repeated painful procedures, which is still perceived even when sucrose is used, compared to placebo. It is concluded that there is a gap in the knowledge about the repeated use of sucrose as a gold standard measure for the management of neonatal pain^23^.

Other pharmacological interventions used less frequently for pain relief were continuous sedation followed by intermittent fentanyl (dose). Continuous intravenous infusion of fentanyl is the most widely used form of administration due to the stability of the therapeutic serum levels of the drug. This form of administration triggers the phenomenon of tolerance though, increasing doses of the medication being necessary to obtain the desired analgesic effect^12^. The efficacy and necessity of using pharmacological interventions to relieve neonatal pain are recognized and necessary in the scenarios that attend to premature neonates, but they have specific indications and undesirable side effects^12^. Local sedation has been used only once, however, but EMLA has shown to be effective for procedures such as venipuncture and lumbar puncture, with few side effects in neonates, more commonly methemoglobinemia and local hyperemia, when used properly^12^.

It is also worth mentioning the low use of maternal skin-to-skin contact, breastfeeding and maternal milk for the relief of acute neonatal pain, interventions considered more natural, with their proven benefits, including Brazilian research^14^ ^-^ ^15^, and that permit the active participation of the mother in care for the child, besides being interventions nursing has autonomy to indicate and use in clinical practice. Especially for premature infants, the skin-to-skin intervention, in which the diaper-only newborn is positioned vertically between the mother’s naked breasts and covered by a sheet or blanket, should be encouraged as, besides providing for mother / baby bonding, the mother influences the pain and stress response of the preterm infant^14^.

Finally, it is important to reaffirm the limits of the comparisons and discussion about the differences in the exposure of preterm infants to potentially painful procedures and interventions for pain relief, due to the methodological differences between the studies, clinical conditions of the neonates and the complexity of the care provided in the neonatal units, which differ in technological density and care protocols. Regardless of these aspects, however, painful procedures occur due to the need for the therapeutic and diagnostic implementation of the infant. Thus, the health team, especially the nursing team, due to their constant contact and closeness to the premature infant, faces a challenge concerning pain management in neonatal services, in order to reduce the exposure of this population to painful procedures, avoiding unnecessary procedures, planning and grouping care, and to use interventions for pain relief.

## Conclusion

It is observed that preterm infants are still submitted to high amounts of painful procedures, the most frequent type of procedure being oral / nasal aspiration. In addition, regarding the contextual factors, it is noted that preterm infants are more exposed to pain according to the birth (sex, birth weight, Apgar, gestational age and chronological age) and clinical conditions (clinical risk score, ventilation support, length of hospitalization and clinical diagnosis). It is also observed that there is under-treatment of the pain resulting from these procedures. Hence, the present study contributes to a more in-depth understanding of pain dimensioning in preterm infants and presents data to support future evidence-based actions to qualify pain management in preterm infants.
